# Discrimination between Sedimentary Rocks from Close-Range Visible and Very-Near-Infrared Images

**DOI:** 10.1371/journal.pone.0132471

**Published:** 2015-07-06

**Authors:** Susana Del Pozo, Roderik Lindenbergh, Pablo Rodríguez-Gonzálvez, Jan Kees Blom, Diego González-Aguilera

**Affiliations:** 1 Department of Cartographic and Land Engineering, University of Salamanca, Polytechnic School of Avila, Avila, Spain; 2 Department of Geoscience and Remote Sensing, Delft University of Technology, Delft, The Netherlands; University of Nebraska Medical Center, UNITED STATES

## Abstract

Variation in the mineral composition of rocks results in a change of their spectral response capable of being studied by imaging spectroscopy. This paper proposes the use of a low-cost handy sensor, a calibrated visible-very near infrared (VIS-VNIR) multispectral camera for the recognition of different geological formations. The spectral data was recorded by a Tetracam Mini-MCA-6 camera mounted on a field-based platform covering six bands in the spectral range of 0.530–0.801 µm. Twelve sedimentary formations were selected in the Rhône-Alpes region (France) to analyse the discrimination potential of this camera for rock types and close-range mapping applications. After proper corrections and data processing, a supervised classification of the multispectral data was performed trying to distinguish four classes: limestones, marlstones, vegetation and shadows. After a maximum-likelihood classification, results confirmed that this camera can be efficiently exploited to map limestone-marlstone alternations in geological formations with this mineral composition.

## Introduction

The knowledge of the mineralogical composition of sedimentary rocks is relevant to many disciplines and sectors. In the fields of Geology and Orogeny to discover this composition is the key for interpreting plate tectonic settings. Many regions have been destroyed and the only record lies in the sediments of the area. Thus, the relationship between the mineralogical composition of sediments and the tectonic plates provides a powerful tool for recognizing ancient tectonic settings [1]. However, ascertaining composition of geological formations in ancient sedimentary basins is generally difficult due to chemical and physical modification of source materials during weathering, erosion, transport and deposition [2]. For other fields such as Environmental Economics, the study of outcrops threatened by human activity and natural hazards as erosion, salinization and landslides is an important contribution. The clay and calcium carbonate contents of the soil are used to describe soil types and reveal their vulnerability to erosion [3, 4].

The past decade has seen the rapid adoption of digital measurement techniques in Geology due to the great advantages that they provide in contrast to expensive traditional techniques. Global navigation satellite systems (GNSS), photogrammetry, laser detection and ranging (LIDAR) and remote sensing satellite imaging systems have all been used for digital mapping and interpretation at multiple scales [5]. Focusing on the field of remote sensing, the shortwave infrared (SWIR) spectral range enable highly effective geological mapping [6], because rocks and minerals have their own inherent spectral pattern in this range [7, 8]. Multispectral satellite data is acquired at 10–30 m spatial resolution. For this resolution, satellite acquired signal in a pixel frequently corresponds to a mixture of several types of ground covers. Close-range remote sensing solves this spatial resolution problem avoiding such mix-up of covers. Collecting high spatial resolution data in a more flexible way and without inadequate temporal resolution due to orbital coverage patterns are some of the advantages of this technique. But it provides many other benefits as several authors [9–11] highlight: the ability to inspection restricted areas and not only the top of the outcrops, allowing real-time registration data for any configuration and orientation of the rock formation. Thus, by analysing close-range remote sensing data acquired from a versatile field-based platform it is possible to obtain more rigorous analysis or even improve the classification of multi-temporal satellite imagery.

Photogrammetric outcrop models [12] provide the framework for geological mapping and interpretation, which become indispensable to perform stratigraphic and structural analysis [13]. Some studies have proven the ability to discriminate between different sedimentary rocks and classify images from some outcropping terrains through the integration of multiple spatial and spectral close-range data. Hyspectral imagery and terrestrial LIDAR data fusion is a good example because they have proven to be a perfect combination to analyse different carbonate-rich outcrops [14–16]. Other works [17] demonstrate the feasibility of lithological interpretations and clay content predictions in sedimentary rocks by analysing the intensity from different wavelength terrestrial laser scanners. For all these works, sensors that cover the SWIR range (1.300–2.600 μm) have been used.

In this article the use of a 6-band multispectral camera covering the VIS and VNIR spectral range (0.530–0.801 μm) is proposed to demonstrate its ability to discriminate sedimentary rocks. A set of 12 geological formations with different percentages of clay and carbonate minerals were studied showing the potential of low-cost sensing for noncontact measurements in this field. In this way, geomorphology, geological mapping, exploration, geochemical hazards and other geological applications could be remotely assessed by using passive sensor technologies at ground level. The paper is organized as follows: Section 2 describes study area where the radiometric campaign took place; Section 3 explains the instruments used for data acquisition, and the data processing in which the protocol followed is also described; Section 4 describes and analyses the results achieved after the data processing, that is, the spectral signatures of different rock types, and the classified multispectral images. Finally, Section 5 includes the conclusions arising from the use of this sensor in this field and the future work.

## Study Area

**Fig 1:**
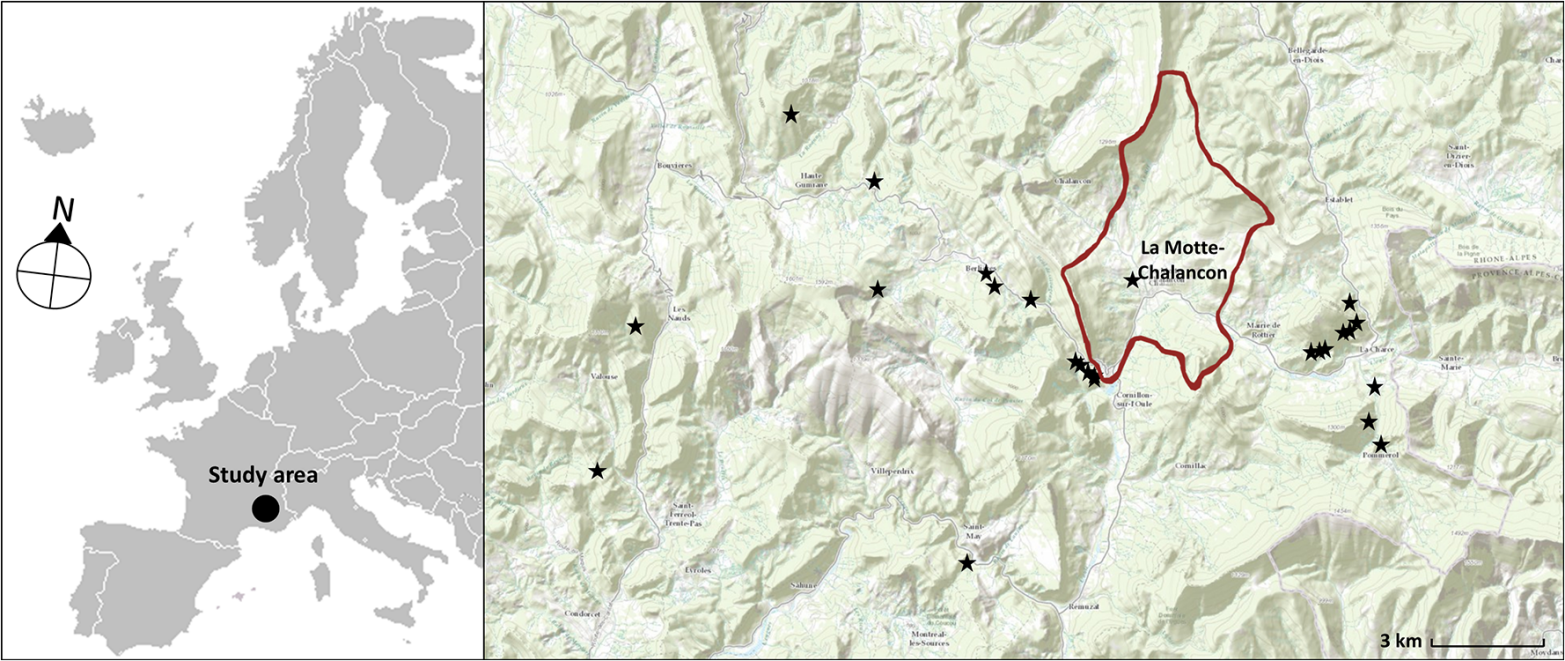
Location of the study area and the studied outcrops (the Drôme department, France).

**Fig 2:**
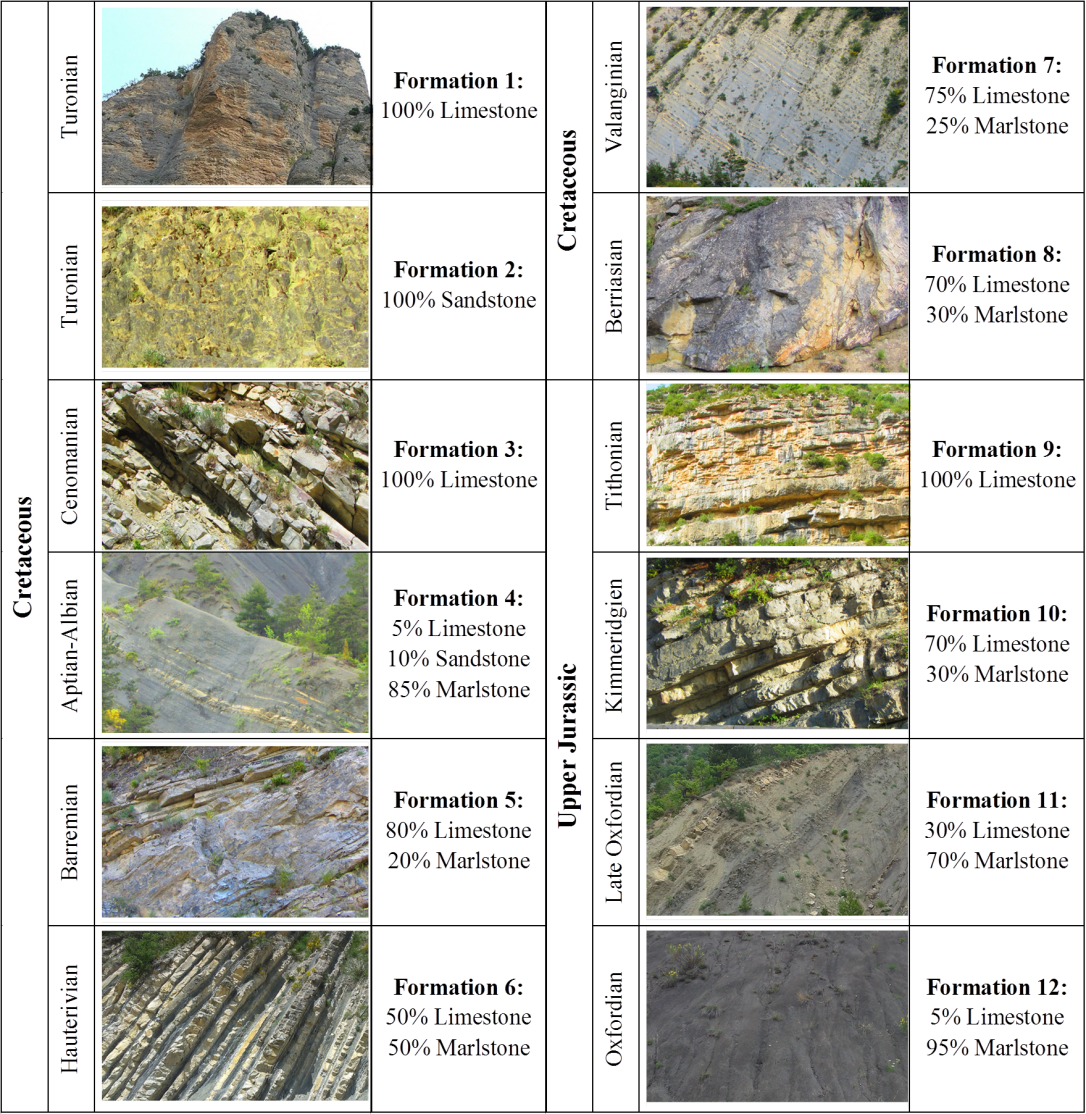
Stratigraphic column of the 12-different Mesozoic geologic formations from newest to oldest.

The radiometric campaign was carried out in June 2014 in the Drôme department of France, in the southeastern part of the Rhône-Alps region (Fig 1). This area is lithologically characterized by sedimentary deposits of the Upper Jurassic-Lower Cretaceous interval. In these periods, carbonate sedimentation was deposited at different water depths: a few meters only of depth in shallow-marine areas [18], and up to several hundred meters of water depth in pelagic marine areas. During these periods there were different deposition processes which gave rise to the current outcrops mainly consisting of carbonate minerals with trace amounts of silica (limestones) and clay minerals (marlstones) with different degrees of uniformity across the layers of strata. On one hand, there were heterogeneous formations with limestone-marlstone alternations formed during the Kimmerdigien, Valanginian or Hauterivian ages (see Fig 2). On the other, there were more homogeneous massive outcrops from the Tithonian and Turonian ages. And finally, marlstone outcrops formed during the Oxfordian, Aptian and Albian ages [19]. The geology, geo-chemistry and mineralization of the study area are well described in the literature [20, 21].

It was possible to identify 12 different geologic formations, which together make up the stratigraphic column of the area (Fig 2). These outcrops are composed of limestone-marlstone alternations, with occasional sandstone beds, with different thicknesses and percentages. In all, the total stratigraphy is made up of three rock types: limestone, marlstone and sandstone, with variations of mineral content and different degrees of weathering. In this regard, the investigation presents some difficulties and challenges due to the mentioned variety of the rock masses.

Since only two of the twelve rock formations, Formation 2 and 4 (Fig 2), were composed of sandstone (100% and 10% respectively), the analysis was mainly focused on the discretization between the other two rock types: limestone and marlstone. In this case eleven of the twelve existing formations were composed of these two rock types.

It was decided to perform a first analysis focusing on the discrimination of the pure formations due to the considerable variety in their composition and the spectral range and resolution limitations of the multispectral camera employed. Formation 1, 3 and 9 were analysed as pure limestones, Formation 2 as pure sandstone and Formation 12 as pure marlstone. Two mixture formations, Formation 4 and 6 were also analysed after a supervised classification. At first sight, and for a non-specialist user in this field, Formation 4 and Formation 12 seem to be the same; so one extra goal was to assess different spectral behaviours of them within the recording spectral rage.

## Material and Methods

### Equipment

**Fig 3:**
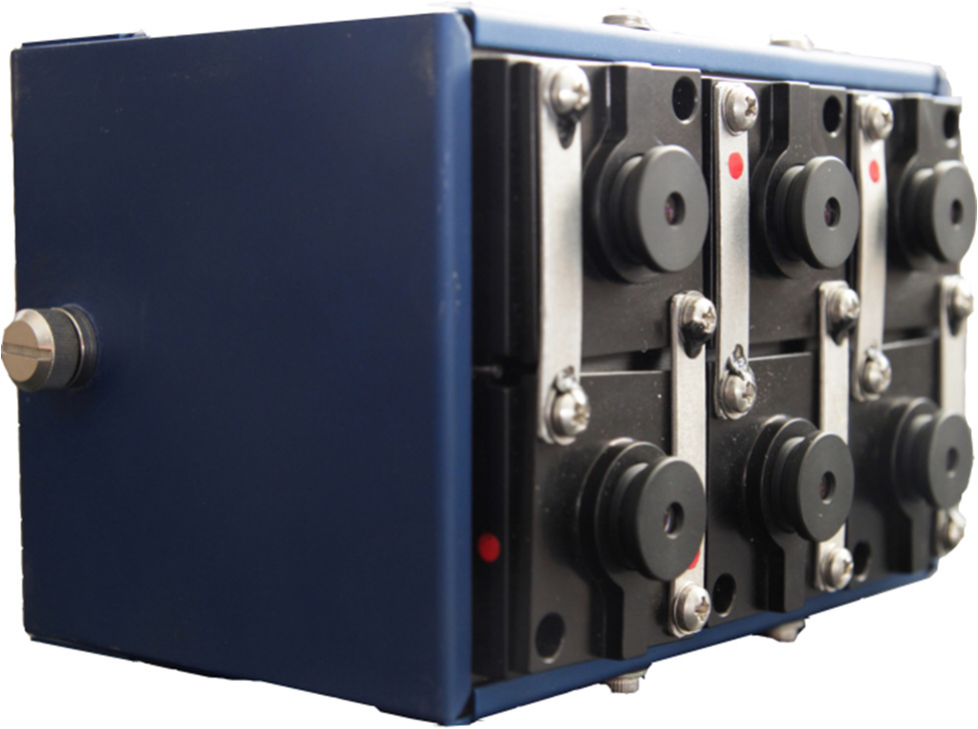
Tetracam Mini MCA-6 multispectral camera.

For the geological data acquisition the Tetracam Mini MCA-6 multispectral camera (Fig 3) and several complementary devices for supporting the data collection were used. The 6-bands multispectral camera is mounted on a special platform and fixed to an adapter swivel to allow getting stable shots during the data acquisition. This sensor consists of six bands covering the visible and very-near infrared range (0.530–0.801 μm) of the spectrum and collects the reflected solar radiation from each rock formation at 10-bits radiometric resolution. Each band is formed by a filter and a CMOS sensor (Complementary Metal-Oxide Semiconductor) providing each band its individual behaviour regarding the captured wavelength and the transmittance. The technical specifications of this sensor are shown in Table 1.

**Table 1:** Characteristics of the Mini MCA-6 multispectral camera.

Features	Bandwidth
Number of bands: 6	Band 1 (0.530 μm): 40 nm
Distance between lenses: 34.5 mm	Band 2 (0.670 μm): 40 nm
Geometric resolution: 1280 x 1024	Band 3 (0.700 μm): 80 nm
Radiometric resolution: 10 bits	Band 4 (0.740 μm): 40 nm
Pixel size: 5.2 μm	Band 5 (0.780 μm): 80 nm
Focal length: 9.6 mm	Band 6 (0.801 μm): 80 nm

**Fig 4:**
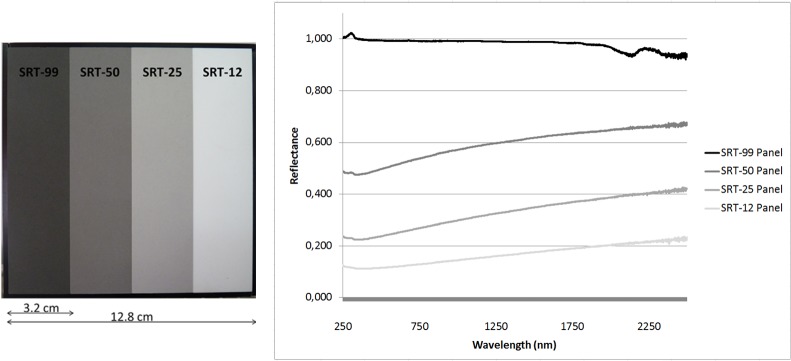
Hemispherical spectral reflectance factor of each Spectralon panel.

In order to obtain images with physical values (reflectance) from raw digital images two essential parameters must be known, the radiometric calibration parameters of each of the six bands (offset and gain, *c0* and *c1*), and the solar irradiance (*E*) of the capture moment. Since the multispectral camera was radiometrically calibrated in a previous field campaign, *c0* and *c1* per band were known [22]. This calibration was a radiometric vicarious calibration based on the radiance method and closely related to the empirical line correction approach [23, 24]. On the other hand, the solar irradiance for each capture moment was obtained by using a standard calibrated reflection target (Spectralon, Labsphere) as will be explained below. The calibrated Spectralon used in this study (Fig 4) consisted of four different panels of 99%, 50%, 25% and 12% reflectance. The spectral behaviour of each Spectralon panel was certified in laboratory.

**Fig 5:**
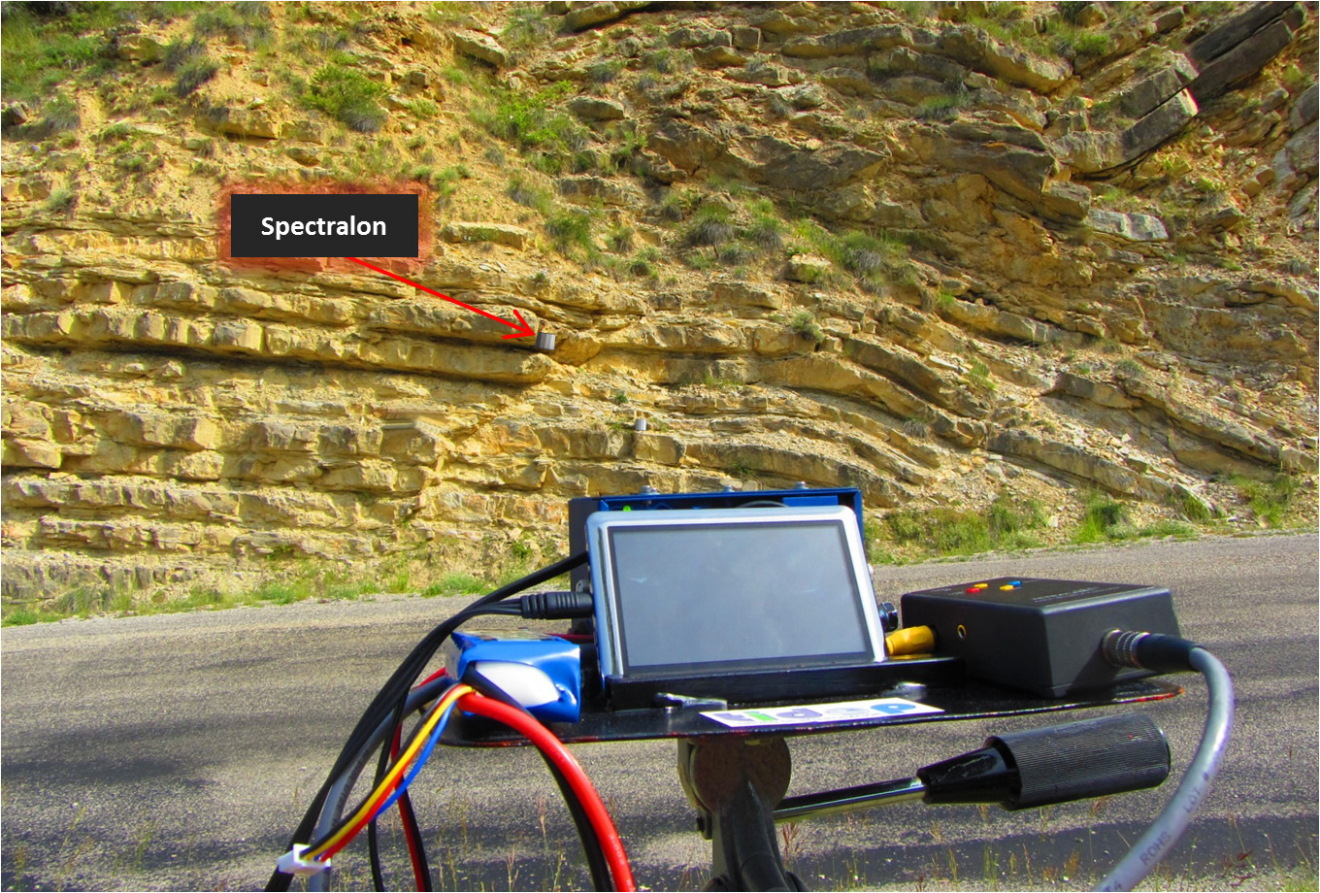
Positioning of the multispectral camera with respect to a rock formation including the Spectralon placed on the wall of the rock.

Finally and because this camera is originally designed to be load on board unmanned aerial systems (UASs), auxiliary equipment becomes necessary for fixing the camera and all its devices to use it at ground level. For this purpose, a special platform to gather all the equipment was designed (see Fig 5). This platform along with a tripod and a swivel provided stability and allowed to rotate the camera in all three degrees of freedom to accommodate and level its position to the orientation of the outcrops.

### Data acquisition protocol

During the field work a total of 40 images were acquired to cover the 12 different geologic formations in different scenarios of light and time of the day in order to have sufficient representative samples under different conditions. Fig 1 shows the exact location of all the outcrops that were sampled around the principal village, La Motte-Chalancon.

At each outcrop considered, the Spectralon was placed on the wall of the rock mass with the same orientation as the exposed surface (Fig 5), after which the ideal place to position the multispectral equipment was determined. Parameters as distance, orientation relative to the face of the rock and sun orientation were taking into account for that purpose. The distance is a significant parameter because both spatial resolution and the parallax between images depend on it. The Ground Sample Distance (*GSD*) is determined as the size of the pixel when the image is projected to the ground surface. The *GSD* value is affected by distance as characterized in Eq (1). Since the six camera lenses are not collinear, there is a parallax that affects images depending on this parameter. The greater the distance, the less parallax, until a limit is reached beyond which the parallax is becoming negligible. This limit is 64 m according to Eq (1). Note that the distance between objectives is 34.5 mm according to the manufacturer (see Table 1). As a consequence, the *GSD* has to be greater than 34.5 mm to avoid significant influence of parallax.

$$GSD=\frac{D\cdot S}{f}$$ (1)

where *D* is the distance between the camera and the outcrop, *S* the pixel size of the sensor and *f* the focal length (see Table 1).

Thus, a balance between spatial resolution and the minimization of the parallax effect was sought. At that distance and with the right orientation thanks to the tripod swivel, visible and very near infrared images were taken such that all the exposed face was covered, whenever possible.

### Data processing

**Fig 6:**
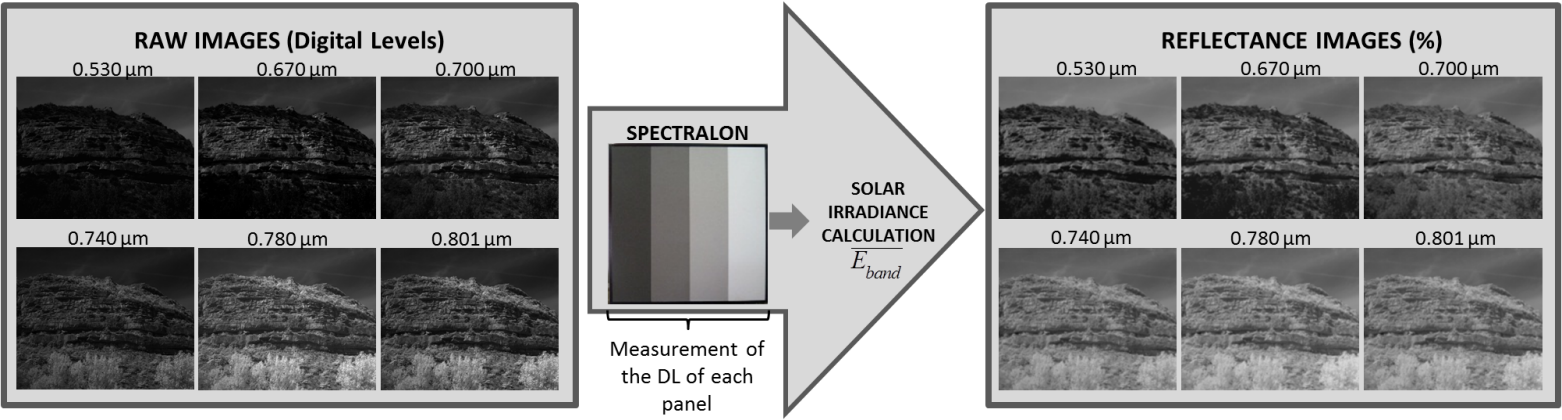
Workflow for obtaining images in reflectance values.

Data processing involved three main phases: obtaining the solar irradiance at the moment of capture, transforming raw digital images into reflectance images (Fig 6) and performing supervised classifications trying to draw conclusions from the composition differences among the geological formation analysed.

Solar irradiance at the moment of capture. Since data were collected at different locations and moments of the day, they were affected by the particular solar radiation at the moment of capture. It is necessary to eliminate the influence of this variability when extracting joint information from the data. In order to obtain images with pixel values independent of sunlight (reflectance images), digital levels (*DL*) of the raw spectral images have to be converted into surface reflectance values. This transformation requires the knowledge of the solar irradiance (*E*) at the precise moment of the image capture and the radiometric calibration parameters of each camera band (*c*0_*band*_ and *c*1_*band*_). The solar parameter was calculated from the reflected solar radiation of a Spectralon (Fig 4). For this purpose, the Spectralon was placed in every outcrop so that it appeared in each multispectral image (Fig 5). In the image processing phase, and making use of its 4 reflectance panels, an average of the raw pixel values panel was determined. Thereby, 4 representative values of each reflectance panel per band (*DL*_(*STR*−99),*band*_, *DL*_(*STR*−50),*band*_, *DL*_(*STR*−25),*band*_ and *DL*_(*STR*−12),*band*_) were estimated. These values, together with the radiometric calibration parameters of each camera band, allowed the radiance values calculation (Eq (2)). The calibration parameters were known beforehand since a vicarious radiometric calibration of the camera [22] was previously performed. A set of 4 radiance values per band (*L*_*i*,*band*_), one for each Spectralon panel, were estimated.

$$L_{i,band}=c0_{band}+\frac{c1_{band}\cdot DL_{i,band}}{T_{band}}$$ (2)

With these parameters and according to Eq (3), an average of the solar irradiance ($\bar{E_{band}}$ [W.m2.sr-1.nm-1]) was estimated for each capture moment and band as an average of the four *DL* values.

$$\bar{E_{band}}=\frac{1}{4}\cdot \sum_{i=1}^{4}\frac{L_{i,band}}{T_{band}}\cdot \pi$$ (3)

where *c*0_*band*_ and *c*1_*band*_ are the radiometric calibration coefficients, of each camera band, *T*_*band*_ is the exposure time per band at each moment of the image capture and *DL*_*i*_ are the digital levels of each reflectance panel of the Spectralon.

#### Reflectance images

After this processing step, the first results of the research, spectral signatures of the outcrops, were obtained. Getting reflectance images involves transforming, digital levels of the raw images into reflectance values. In this way, assuming the outcrop as a rocky Lambertian surface (uniform reflectivity), the following Eq (4) is applied to every pixel of each six multispectral images.

$$R_{band}{}_{\left(\%\right)}=\frac{c0_{band}+c1_{band}\cdot DL}{\bar{E_{band}}}\cdot \pi$$ (4)

where *R*_*band*(%)_ is the reflectance value of each pixel in percentage. *c*0_*band*_, *c*1_*band*_ and $\bar{E_{band}}$ are the calibration parameters of the camera and the solar irradiance, respectively.

**Fig 7:**
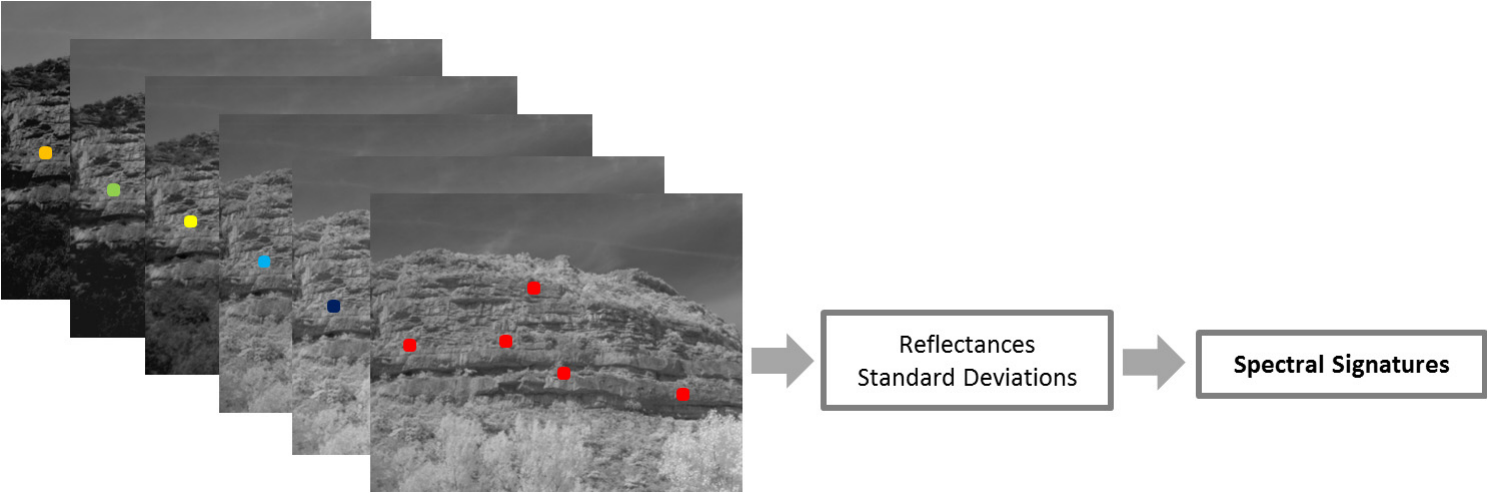
Reflectance per band of a specific rock formation.

**Fig 8:**
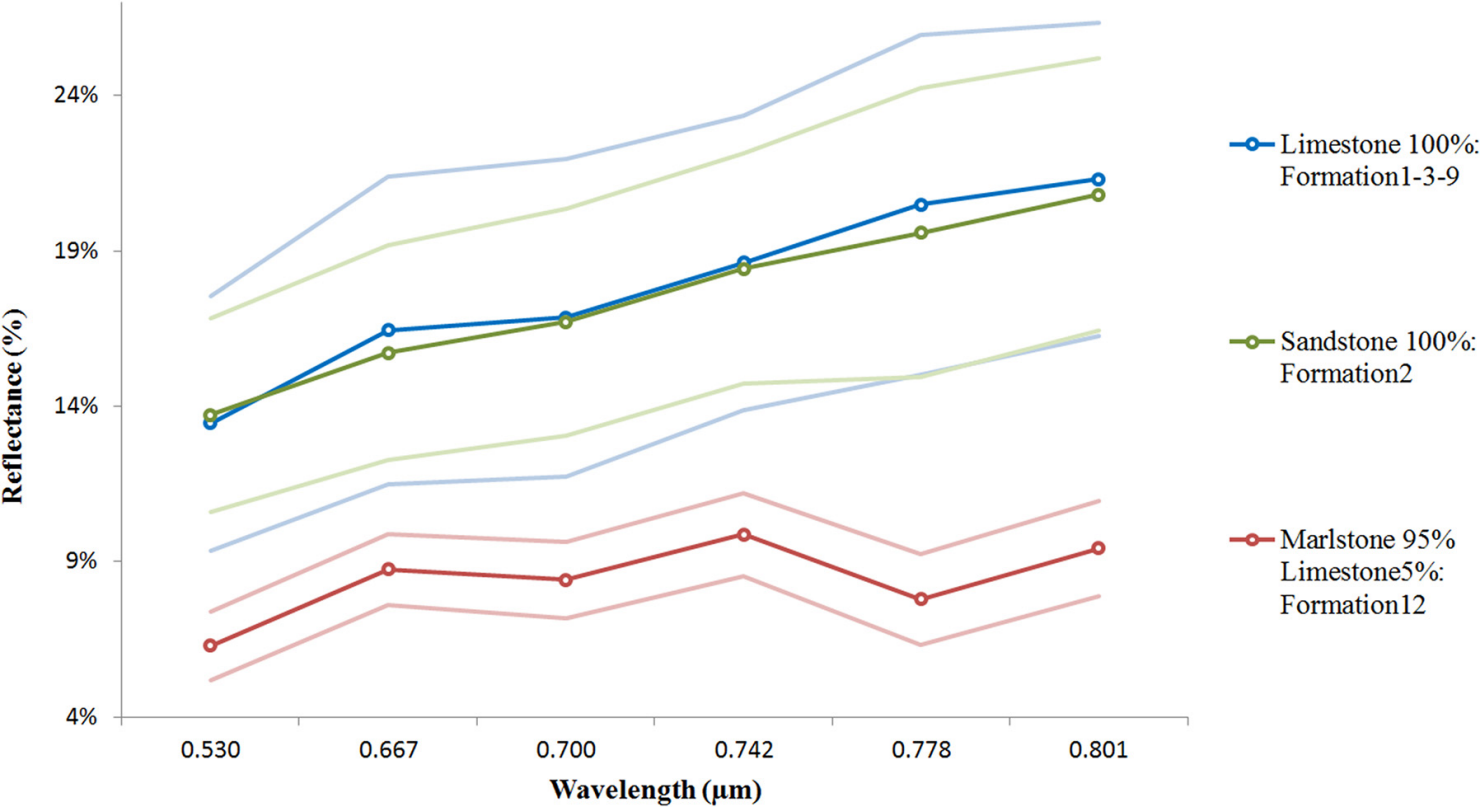
Spectral signatures of the three rock types and the standard deviation of their measurements.

Once the reflectance images were obtained, multispectral images were created and stored as 6-dimensional matrixes (1024 pixels x 1280 pixels x 6 images) where reflectance values per band can be extracted just by clicking a pixel (Figs 7 and 8). If these reflectance pixel values are plotted on the y-axis and the respective wavelengths of the camera on the x-axis, spectral signatures of each geological formation are obtained. Taking into account the mean values and standard deviations of the outcrops, conclusions about the discrimination potential of the camera in this field were derived.

#### Classified images

The third step classifies the multispectral images into 4 classes: limestone, marlstone, vegetation and shadows; resulting in an easy-to-interpret classified image that will help to assess the use of this sensor in the characterization and recognition of rocks. For this purpose, a supervised classification based on the maximum likelihood (ML) algorithm [25] was performed after masking image pixels belonging to sky and/or road.

The ML classifier quantitatively evaluates both the variance and covariance of the category spectral response patterns when classifying an unknown pixel (*x*). Four different sets were prepared to train the process assuming that the distribution of the pixels forming the category training data is Gaussian. This assumption of normality is generally reasonable for common spectral response distributions. Under this assumption, the distribution of a category response pattern (*k*) can indeed be completely described by the mean vector and the covariance matrix. With these parameters, the statistical probability of a given pixel value being a member of a particular land cover class (*P*(*x* / *k*)) can be computed. The resulting bell-shaped surfaces are called probability functions (Eq (5)), and there is one such function for each spectral category [26].

$$P(x/k)=-\text{ln}\;\left|\sum k\right|-{\left(x-\mu_{k}\right)}^{T}\sum k^{-1}(x-\mu_{k})$$ (5)

where |∑*k*| is the covariance matrix and *μ*_*k*_ the mean vector of the training data belonging to each class.

In this way, a class is assigned to a pixel when the probability of belonging to this class in Eq (5) is maximal.

## Experimental Results

### Spectral signatures

This research covers the study of 12 geological formations composed of three types of sedimentary rocks, sandstone, limestone and marlstone. Fig 8 depicts the spectral signatures of the geological formations with higher percentages of each of these rocks: Formation 1, Formation 2, Formation 3, Formation 9 and Formation 12 (compare Fig 2). Formation 1, 3 and 9 were analysed as pure limestones, Formation 2 as pure sandstone and Formation 12 as pure marlstone. For each rock formation manually representative pixels were selected, sampling areas clear of vegetation and shadows. For each selected pixel, reflectance values of the 5x5 nearest neighbours (involving 25 pixels) were stored. Finally, the average reflectance and standard deviation of the stored values were calculated with the support of software developed using Matlab.

For the considered samples the lowest standard deviation value (with an average of 1.3%) appears for the Formation 12 (95% marlstone). This behaviour is consistent with the hypothesis that experts support, errors in spectral measurements increase when the grain size increases [27]. A large grain has a greater internal path where photons may be absorbed [28]. Indeed marlstones have the smallest grain size of the three types of rocks (< 0.06 mm) due to the clay minerals composition [29]. On the other hand, the highest deviation value occurred for limestones (with an average of 4.5%) very close to sandstones (with an average of 3.6%). In this case, there is much variability in the grain size due to the different composition of its fragments; but also because the spectral signature of limestones was calculated as an average of three different formations. Finally, another source of variability in the reflectance of a rock is the degree of mechanical weathering [30], so depending on the massivity and the degree of homogeneity of the formation more or less errors were obtained in the measurements.

By analysing the reflectivity of the examined formations it can be confirmed that the spectral signatures are coherent with the reflectance behaviour of their minerals. Eleven of the twelve geological formations are composed of different proportions of limestone and marlstone. Only Formation 2 and 4 are composed of sandstone (100% and 10% respectively). For that reason, the analysis was mainly focused on the discretization between these two rock types (limestones and marlstones). Limestones are composed mainly of calcium carbonate. Moreover, marlstones are composed of clay. Due to the reflective properties of these materials (higher reflectance in the case of calcium carbonate [31]) depending on the percentage of these components, more or less reflectance was obtained. Fig 8 shows that the spectral signature of sandstones and limestones in the 0.530–0.801 μm spectral range is quite similar. In addition, Fig 8 indicates that it is possible to discriminate between limestones and marlstones because, in spite of the high variability of limestone measurements, both spectral signatures do not overlap.

**Fig 9:**
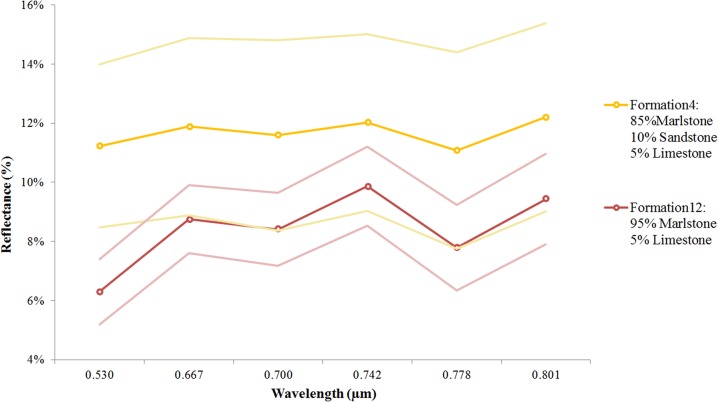
Spectral signatures of Formation 4 and 12 and the standard deviation of the measurements.

As pointed out in Section 2, obtaining any difference between Formation 4 and Formation 12 was a challenge because these formations, at first sight, look the same. As shown in Fig 9, and despite of the reflectivity differences between these two formations, we may not be able to obtain good results from a classification based only on these two formations due to their spectral patterns overlap (taking into account the standard deviations). Nevertheless, their spectral signatures are consistent with their composition; Formation 4 has more reflectivity because it contains sandstone. Regarding the deviation degree of measurements (an average of 3.1% in Formation 4), the explanation lies in the fact that the greater variability in the composition, the greater deviation in the measurements.

### Classification results

**Fig 10:**
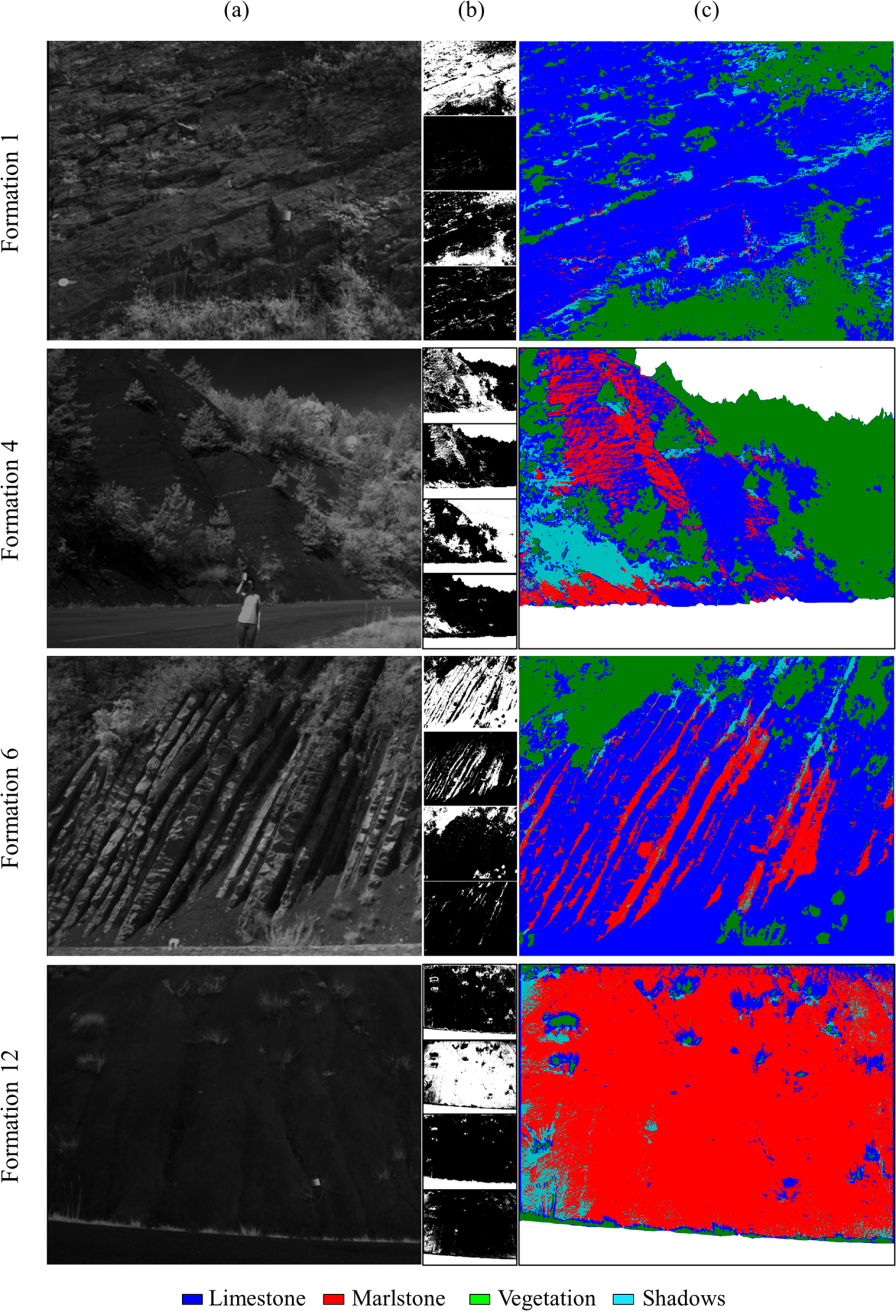
(a) Band-5 reflectance images. **(b) Images of probability**. **(c) Final classified images**.

In the third step of data processing a supervised classification was performed. Four different training sets were chosen to classify final reflectance images into 4 classes: limestone, marlstone, vegetation and shadow. Sandstone was excluded from the classification process as only two formations, Formation 2 and 4, were composed of this material and because the results in Fig 8 indicate that the spectral response of sandstone in the spectral range covered by the sensor is almost identical to that of the pure limestone formation. Formation 1, 3 and 9 were selected to represent pure limestone and Formation 12 representing pure marlstone. Fig 10 illustrates the resulting classified images for the case of four geological formations, pure limestone and marlstone (Formation 1 and Formation 12) and two mixed formations (Formation 4 and Formation 6). In the classified images white pixels represent pixels masked previously to be out of this process.

The classified images were obtained after calculating the probability of belonging to each of the 4 classes. This probability was graphically represented for each class as an 8-bit grey scale image where the maximum probability was represented in white (value of 255) and the minimum in black (value of 0). These probability images are shown in order in Fig 10B: probability of belonging to the limestone, marlstone, vegetation or shadow class. It is observed that vegetation areas are perfectly discriminated except in the case of the Formation 12 where some vegetation areas were classified as limestone. This fact happens due to the pre-set configuration of the camera filters. The wavelengths of each filter were chosen for previous vegetation studies. Vegetation absorbs red and reflects green and infrared radiation. So by analysing red and infrared wavelengths we can provide information about the vegetation´s health [32].

Regarding the mixed formations, in the case of Formation 4 there was a higher percentage of blue pixels (limestone) even though their real percentage is 5%. A possible explanation was the presence of sandstone (10%) and because the slope of the outcrop influenced the way the light was reflected.

To evaluate the separability between classes the transformed divergence based on means and variance-covariance matrix [33] was used as a quantitative estimator for the 4 training samples. Table 2 shows the separability between the 4 classes (without considering the background or null class).

**Table 2:** Transformed divergence (0–2).

	Limestone	Marlstone	Vegetation
Marlstone	1.999834	-	-
Vegetation	1.999739	2.000000	-
Shadow	1.917816	1.808445	1.988555

As Table 2 shows, results from class separability confirm that the main goal of this study has been achieved, a high separability between limestone and marlstone (99.99%). It must be pointed out that for this results a proper radiometric calibration of the multispectral camera and different lenses corrections [22] were applied to work in reflectance values (characteristic values of each material). The worst case was for marlstone and shadows (90.42% of separability) but remained within an acceptable range. This fact would be explained due to the low reflectivity of this sedimentary rock. In this way, there were some areas where the classification was less efficient than it should be. To conclude, it is also worth noting that whenever vegetation was compared to another class, separability results were quite good. This was expected because the configuration of the wavelengths makes the camera ideal for vegetation studies.

## Conclusions

In this paper a visible-very near infrared multispectral camera was tested and analysed in a field campaign in the Drôme department of France. As a result, its ability and limitations to discretize sedimentary rock formations were evaluated regarding the spectral range of the camera (0.530–0.801 μm) and the homogeneity of the rock surfaces.

Regarding the spectral signatures of the most pure geological formations (Fig 8) it is concluded that although it is not possible to completely discriminate between all of them, the spectral signatures are consistent with their composition. The highest reflectivity response was obtained for sandstones and limestones, which usually consist of quartz and calcite grains respectively. Finally Formation 12, composed mostly of marlstones, obtained the lowest reflectivity. Due to the percentage of sandstone in the Formation 4 (10%) it had higher reflectance than Formation 12 although both mostly had the same grain size and similar degree of weathering.

As these geological formations were mainly composed by 2 of the 3 types of sedimentary rocks analysed (limestone and marlstone), a maximum likelihood supervised classification was performed by distinguishing 4 classes: limestone, marlstone, vegetation and shadows. After comparing the classified images (Fig 10) with their corresponding geological formation composition (Fig 2), it is possible to conclude that this multispectral camera is able to discriminate between these 2 types of sedimentary rocks. It has been demonstrated that limestones and marlstones have different spectral patterns in the 0.530–0.801-μm spectral range. By contrast, it is not possible to find notable differences between the response patterns of limestones and sandstone. The spectral range of this camera does not allow the discrimination between them. It would be possible by using a capture sensor that works in the SWIR range of the spectrum (1.4–3μm).

Derived from this analysis, some conclusions are listed on the difficulties we found in the radiometric analysis of rocks in general and the limitations arising from the use of this camera in particular:

- The orientation of the outcrop complicates radiometric analysis because different orientations and roughness reflect light in different directions [34]. Even if the camera is properly stationed, it will pick different reflectances from the same rock due to the different orientation of their faces.
- The homogeneity of the geological formation is a relevant property. In this study the most homogeneous lithological mass belongs to the Formation 12 [35], the “Terres Noires” formation, which had the lowest standard deviation in its measurements.
- Greater homogeneity in sunlight results in a better radiometric analysis because the rock will be evenly illuminated without the presence of shadows or glare in different parts. Therefore, cloudy days are the most suitable days for data collection.
- The spectral range of the camera is not the most suitable for characterizing different types of geological formations, although results in the discrimination of rock types are encouraging.

Future improvements in methods and equipment will help to achieve better results. With respect to methods, Bidirectional Reflectance Distribution Function (BRDF) studies could be incorporated to study how light is reflected at each geological formation and moment. In this way, reflectance results will improve as it is no longer necessary to assume that the surfaces scatter in a Lambertian way and depend on the slope of the outcrop. Regarding the equipment, the combined use of different remote sensors such as terrestrial laser scanners, will complement spectral information and will provide 3D models giving scale, slope and surface roughness. This information may help improve the final classification process.
